# ADVANCE CARE PLANNING ACROSS SOCIODEMOGRAPHIC CHARACTERISTICS AND COGNITION LEVELS

**DOI:** 10.1093/geroni/igad104.1365

**Published:** 2023-12-21

**Authors:** Zahra Rahemi, Swann Adams

**Affiliations:** Clemson University, Clemson, South Carolina, United States; University of South Carolina, Columbia, South Carolina, United States

## Abstract

Older adults from underrepresented and minority groups have higher rates of chronic disease and cognitive impairment and lower rates of advance care planning and comfort care at the end of life. This study examined advance care planning in U.S. older adults across different sociodemographic characteristics and cognition levels. Secondary data analyses were conducted using the 2014 Health and Retirement Study. Logistic regression models were run with each outcome variable (having living will, durable power of attorney for healthcare [DPOAH], and both the living will and DPOAH) stratified by cognition (dementia/impaired cognition versus normal cognition). Prediction variables included race, ethnicity, rurality, marital status, gender, education, age, everyday discrimination, social support, and loneliness. Of the 17,698 respondents, 77.8% had normal cognition and 22.2% had a diagnosis of dementia or impaired cognition. Black and Hispanic participants and younger individuals with lower levels of education were less likely to have a living will or both DPOAH and living will in place. Race and ethnicity were significant predictors of DPOAH among the normal cognition group but not in the dementia group. Rurality significantly predicted having a living will and both DPOAH/living will for the normal cognition group but not the dementia/impaired cognition group. This study provides evidence of the need to understand advance care planning disparities among older adults with multiple marginalized identities and different cognition levels. The findings are important for examining advance care planning regarding what matters to diverse older adults as they age and confront health and cognition decline.

